# Time trends in treated incidence, sociodemographic risk factors and comorbidities: a Finnish nationwide study on anxiety disorders

**DOI:** 10.1192/j.eurpsy.2022.209

**Published:** 2022-09-01

**Authors:** P. Khanal, T. Ståhlberg, T. Luntamo, D. Gyllenberg, K. Kronström, A. Suominen, A. Sourander

**Affiliations:** University of Turku, Research Center For Child Psychiatry, Turku, Finland

**Keywords:** Anxiety disorders, treated incidence, risk factors, comorbidities

## Abstract

**Introduction:**

Anxiety disorders (ADs) are common in childhood and adolescence and global estimates suggest they affect 6.5% of individuals under 19 years of age.Yet, there has been a lack of research on time trends and socio-demographic risks for children and adolescents who receive treatment for ADs.

**Objectives:**

We aim to fill gaps in our knowledge by examining a nationwide sample of Finnish children and adolescents diagnosed in specialized healthcare settings.

**Methods:**

We used register data of all singleton children born in Finland from 1992-2006 and diagnosed with ADs from 1998-2012. Changes in time trends in incidence were studied by dividing the study sample into three cohorts by birth years: 1992-1996, 1997-2001 and 2002-2006. The 22,388 individuals with ADs were matched with 76,139 controls. Nested case-control design was used to study the socio-demographic risk factors.

**Results:**

Comparing the 1992-1996 and 2002-2006 cohorts showed the cumulative incidence of treated ADs at the age of 10 increased from 0.3% to 1.2% (females) and 0.46% to 1.9% (males). Subjects had higher odds of being diagnosed with an AD if mothers had low SES (OR 1.49, 95% CI 1.42-1.58) and were single parents (OR 1.99, 95% CI 1.84-2.15) at birth. Unipolar depression was the most common psychiatric comorbidity (31.2%).

**Conclusions:**

ADs diagnosed by specialized services increased from 1998-2012 in both genders. This could indicate real increase in overall ADs, an increase in seeking treatment or both phenomena. The findings on maternal socioeconomic status and single parenting help improve understanding of environmental risk for anxiety disorders among children and adolescents.

**Disclosure:**

No significant relationships.

